# High-Intensity Focus Ultrasound Ablation in Prostate Cancer: A Systematic Review

**DOI:** 10.3390/jpm14121163

**Published:** 2024-12-20

**Authors:** Che-Hsueh Yang, Daniela-Viviana Barbulescu, Lucian Marian, Min-Che Tung, Yen-Chuan Ou, Chi-Hsiang Wu

**Affiliations:** 1 Department of Urology, Changbing Show Chwan Memorial Hospital, Changhua 505, Taiwan; b101098093@tmu.edu.tw; 2 Department of Medical Oncology, ONCOHELP Hospital, 300239 Timisoara, Romania; danielaviviana53@gmail.com; 3 Department of Urology, “Pius Brînzeu” County Emergency Clinical Hospital, 300723 Timisoara, Romania; lucimarian93@gmail.com; 4 Division of Urology, Department of Surgery, Tungs’ Taichung MetroHarbor Hospital, Taichung 435, Taiwan; t1142@ms.sltung.com.tw

**Keywords:** prostatic neoplasms/surgery, neoplasm recurrence, local/surgery, treatment outcome, ultrasound, high-intensity focused, transrectal/methods

## Abstract

**Background/Objectives:** Prostate cancer (PCa) outcomes vary significantly across risk groups. In early-stage localized PCa, the functional outcomes following radical prostatectomy (RP) can be severe, prompting increased interest in focal therapy, particularly High-Intensity Focused Ultrasound (HIFU). This study is to summarize the current clinical trials of HIFU on PCa. **Methods:** We reviewed clinical trials from major databases, including PubMed, MEDLINE, Scopus, and EMBASE, to summarize the current research on HIFU in PCa treatment. **Results:** The literature highlights that HIFU may offer superior functional outcomes, particularly in continence recovery, compared to RP and radiation therapy. However, the oncological efficacy of HIFU remains inadequately supported by high-quality studies. Focal and hemigland ablations carry a risk of residual significant cancer, necessitating comprehensive patient counseling before treatment. For post-HIFU monitoring, we recommend 3T magnetic resonance imaging (MRI) with biopsy at 6 to 12 months to reassess the cancer status. Biochemical recurrence should be defined using the Phoenix criteria, and PSMA PET/CT can be considered for identifying recurrence in biopsy-negative patients. **Conclusions:** Whole-gland ablation is recommended as the general approach, as it provides a lower PSA nadir and avoids the higher positive biopsy rates observed after focal and hemigland ablation in both treated and untreated lobes. Future study designs should address heterogeneity, including variations in recurrence definitions and surveillance strategies, to provide more robust evidence for HIFU’s oncological outcomes.

## 1. Introduction

According to the American Cancer Society (Atlanta, GA, USA; available at https://www.cancer.org/cancer/types/prostate-cancer/about/key-statistics.html (accessed on 2 August 2024)), the estimated new cases and deaths of prostate cancer (PCa) in 2024 were approximately 300,000 and 35,000, respectively. Since the changes in screening recommendations in 2014, incidences have increased by 3% annually. As a result, PCa has become the second-most common cause of cancer-related deaths, with approximately 1 in 45 men affected. PCa exhibits widely varying outcomes, ranging from lethal in some cases to asymptomatic in others. In the United States, approximately 3.3 million men diagnosed with PCa are alive without requiring advanced treatment. According to the Surveillance, Epidemiology, and End Results (SEER) program, about 70% of PCa cases are initially diagnosed as localized, with a 5-year relative survival rate exceeding 99% (available at https://seer.cancer.gov/statfacts/html/prost.html (accessed on 2 August 2024)). In China, PCa ranked as the 12th most common cancer in 2005 and the 8th in 2020. Over this period, the prevalence increased by approximately 5% in all-age deaths, with over half of this attributed to an aging population [1]. While the overall incidence rate has risen, the incidences of high-grade localized PCa have significantly declined [2]. Conversely, low-grade localized PCa has increased significantly, at an annual rate of 3%, compared to the annual 10% decrease in high-grade disease [2]. In our experience [3], among men without prior biopsy, approximately 85% were diagnosed with localized PCa of Gleason grade (GG) group ≤ 2, and only about 10% had T-stage > 3a. This suggested that the typical presentation of newly diagnosed localized PCa involves PSA levels around 10 ng/mL, GG group ≤ 2, and T-stage < 3a. Most of these cases fall into the favorable intermediate-risk group or lower. Under such conditions, active surveillance (AS) or observation is recommended by the National Comprehensive Cancer Network (NCCN) guidelines, particularly for those with an expected survival of 5 to 10 years [4]. However, AS may yield inferior oncological outcomes in certain patient groups. A randomized controlled study with a 15-year follow-up [5] enrolled men with localized PCa, including 94% with GG group ≤ 2, 90% with PSA ≤ 10 ng/mL, and all with clinical stage ≤ T3a. Although cancer-specific mortality did not differ significantly from radical therapy, the rates of progression and metastasis were significantly higher [5].

Radical treatments, including radical prostatectomy (RP) and radiation therapy, offer excellent survival outcomes for low-risk and favorable intermediate-risk patients. However, the functional impairments can be distressing for both patients and urologists. Clinical trials have reported that up to 50% of men undergoing robot-assisted radical prostatectomy (RARP) for localized PCa continue to use more than one pad per day at 6 months post-surgery [6], with prevalence rates as high as 60% in some reviews [7]. In our experience, approximately 30% of patients experienced persistent incontinence despite surgery performed by skilled surgeons [3]. Regarding potency recovery, the documented rates are 33.6% in randomized controlled trials (RCTs) and 66.4% in non-RCTs [8]. In our experience, around 40% of men reported erectile dysfunction following RARP [3]. In cases of low- and intermediate-risk localized PCa treated with radiation therapy, erectile and continence functions seemed stable in low-risk groups, while erectile recovery in intermediate-risk patients was significantly poorer than in those undergoing RP [9]. To address these challenges, focal therapy has emerged as a strategy to balance oncological and functional outcomes. The NCCN guidelines list several focal therapy devices for PCa treatment, including High-Intensity Focused Ultrasound (HIFU) [4]. HIFU is recommended for non-metastatic recurrent PCa after definitive radiation therapy. Prospective studies have also investigated HIFU for early-stage localized PCa. According to NCCN guidelines, HIFU demonstrated a 5-year failure-free survival rate of 88%, with potency and continence recovery rates of 78% and 98%, respectively [4].

The development of HIFU technology can be traced to the end of World War I when the potential of ultrasound energy was discovered during submarine research. It was observed that ultrasound devices could kill fish along their energy paths [10]. Further experimentation revealed that acoustic cavitation caused skin damage in fish when exposed to therapeutic ultrasound (≤1.0 W/cm^2^, 1 MHz) for 90 s [11]. HIFU focuses ultrasound energy to generate localized heat, producing coagulative thermal necrosis within a millimeter-sized target zone without damaging the surrounding tissues. The ablated area was approximately 1 mm in diameter and 1 cm in length [12]. This phenomenon, known as the “popcorn effect”, is observable in real-time on HIFU device monitors [13,14].

Compared to other ablative techniques like cryotherapy and radiofrequency, HIFU offers superior intraoperative image monitoring, tissue penetration, coagulation, and thermal energy control [15]. The thermal destruction zone in HIFU-treated tissue is highly localized, with a margin of only ~50 μm from adjacent healthy tissue [16]. HIFU has been applied to various solid tumors, including pancreatic [17], liver [18], renal [18], breast [19], and uterine tumors [20]. However, for localized PCa, available oncological and functional data suffer from limitations such as heterogeneous study endpoints, lack of long-term data, and inconsistent surveillance methods. Consequently, HIFU is not officially recognized in PCa treatment guidelines. This review aims to summarize the current understanding and advantages of HIFU in managing early-stage localized PCa.

## 2. Materials and Methods

**Figure 1:**
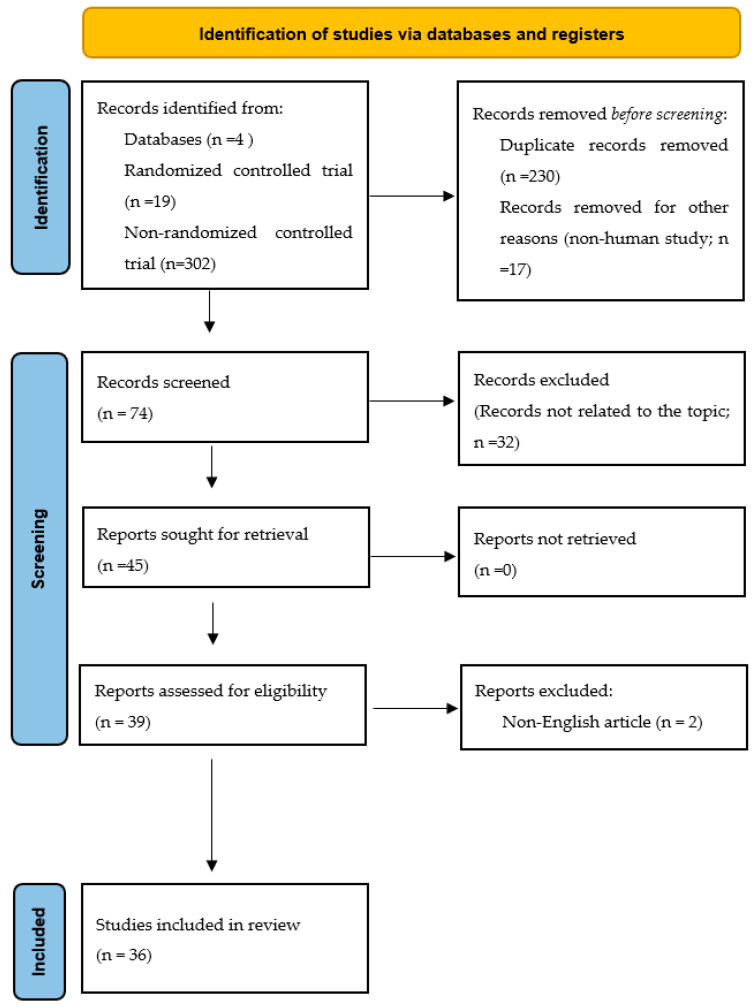
PRISMA guidelines used to screen the articles.

We searched the published literature in PubMed, MEDLINE, Scopus, and EMBASE using the term “(prostate cancer) AND ((High Intensity Focused Ultrasound) OR (HIFU))”. The PRISMA guidelines were applied to screen the articles (Figure 1). This study is not registered in the database. A manual web search was conducted by three authors aiming to collect clinical trials published in English. Case reports, case series, conference abstracts, retrospective studies, and review articles were excluded. Two authors were responsible for screening the abstracts, and the corresponding author provided opinions when discrepancies or uncertainties arose. The main extracted data included basic patient information, follow-up, complications, oncological outcomes, and functional outcomes.

After screening and appraising the literature abstracts retrieved with the aforementioned term, we identified three RCTs [21,22] and 34 phase 2 clinical trials [23,24,25,26,27,28,29,30,31,32,33,34,35,36,37,38,39,40,41,42,43,44,45,46,47,48,49,50,51,52,53,54,55,56]. During the identification process, some articles were categorized as technique development or animal studies and were excluded from further screening (*n* = 24). During the screening process, articles unrelated to our aim, such as those focused on cancer detection or prediction, were also excluded from the final appraisal (*n* = 27). Additionally, two articles were excluded because no English version was available [57,58]. The publication years of all the qualified articles ranged from 1997 to 2023.

## 3. Results

### 3.1. HIFU as Salvage Therapy

Currently, the only recognized role of HIFU in the NCCN guidelines [4] is its salvage use for non-metastatic recurrence following definitive radiation therapy. The oncological outcomes were primarily derived from one clinical trial [51]. The median biochemical recurrence (BCR)-free survival was 63 months, while the 5-year overall survival and cancer-specific survival rates were 88% and 94%, respectively. The published clinical trial data on HIFU for non-metastatic recurrent prostate cancer (PCa) after definitive radiation therapy are summarized in Table 1.

In one study, HIFU was applied as salvage therapy to patients who had previously undergone RP [55]. In the remaining studies, salvage HIFU was used on patients who had received external beam radiation therapy or brachytherapy [50,51,52,53,54]. Regarding the definition of PSA failure after HIFU, the Phoenix criteria were used in four studies [50,52,53,54], while a combination of the Phoenix and Stuttgart criteria was applied in one study [51]. One study did not adopt any criteria to define PSA failure [55]; instead, it categorized successful treatment dichotomously, using a PSA threshold of 0.4 ng/mL. Three studies [50,53,54] performed regular transrectal ultrasound-guided biopsies to confirm disease status at 3 or 6 months postoperatively, while the other three studies [51,52,55] relied solely on PSA monitoring.

In the literature on salvage HIFU for non-metastatic recurrent PCa after radiation therapy, the PSA nadir typically dropped within 3 to 6 months after HIFU. It was observed that ablative strategies other than whole-gland ablation were associated with higher median PSA nadirs [51,52]. Regarding functional outcomes, erectile function scores tended to decline at 6 months post-HIFU, and the reported pad-free continence rates ranged from 50% to 90%. The most common postoperative complication was urinary tract infection or epididymitis. Pubic bone osteitis occurred in 1% to 4% of cases, and the development of fistulas was also reported. Other minor complications, such as strictures and anorectal fistulas, suggested that significant inflammatory reactions and fibrosis could still occur after HIFU.

### 3.2. HIFU in the Localized PCa

In Table 2, we summarized the studies reporting oncological outcomes (event-free survival) and functional outcomes as mentioned in the content. Two studies [26,37] were considered to share the same cohort data, and therefore, only one of them [26] was included in Table 2. Among all the included literature, only nine studies provided sufficient information on oncological outcomes. Of these nine studies, only two [26,41] involved high-volume recruitment. The remaining studies reported short-term outcomes after HIFU, such as 6- or 12-month biopsy results or magnetic resonance imaging (MRI) reevaluation findings.

In the literature on HIFU treatment for localized PCa, 80% to 90% of the included patients were categorized as low- or intermediate-risk groups (GG < 4) and had clinical stages without extracapsular extension or seminal vesicle invasion.

Regarding oncological outcomes, the BCR-free survival rate was worse in patients from higher-risk groups and in those undergoing ablation strategies other than whole-gland ablation. It was noted that approximately 35% of patients had positive findings in intraoperative biopsies after hemi-ablation, indicating that, even with thorough pre-HIFU evaluations, certain limitations persisted. Regardless of the imaging modalities or biopsy techniques used, it is impossible to completely rule out cancer in a specific lobe prior to HIFU. Urologists should communicate this false-negative risk during preoperative assessments before deciding on focal or hemi-ablation strategies.

In terms of functional outcomes, continence was generally well preserved, with the rate of de novo incontinence typically below 10% [29,30,46,56]. However, potency preservation was less favorable, with the incidence of new-onset erectile dysfunction reaching as high as 30–40% [46,56].

### 3.3. Salvage Radical Prostatectomy After HIFU

Two studies reported results regarding salvage RP for HIFU-recurrent PCa [44,47]. One study [44] included a small sample size (*N* = 4) for patients who underwent HIFU treatment, but it did not specify the actual outcomes of the HIFU group. Instead, most of the data in this study pertained to patients previously treated with radiotherapy or brachytherapy. As a result, only one study [47] was included in Table 3.

In the study listed in Table 3 [47], salvage RP was performed via transperitoneal access, with either unilateral or bilateral nerve-sparing techniques. Functional outcomes were assessed using standardized questionnaires. A patient experiencing BCR after salvage RP was found to have pathological features of Gleason Group 4, T3a stage, and lymph node involvement [47]. However, the PSA level of a patient with a positive surgical margin remained at 0.04 ng/mL for 12 months after surgery. Among prostate specimens from salvage RP, 10 cases (76.9%) demonstrated out-of-field recurrent PCa following prior HIFU treatment. Regarding side effects, most serious Clavien-Dindo grade (>III) complications were related to surgical technical challenges, such as anastomotic leakage, fascial dehiscence, and lymphedema.

## 4. Discussion

### 4.1. HIFU in Non-Metastatic Recurrent PCa After Radiation Therapy

According to the statistics, approximately 30% of PCa patients choose radiation therapy as their initial treatment [59]. For localized PCa, radiation therapy has been shown to improve overall survival, even in elderly patients [60]. In cases of recurrent PCa after radiation therapy, treatment options include salvage RP, androgen deprivation therapy (ADT), cryotherapy, brachytherapy, or HIFU. However, due to a lack of high-quality data, no single modality has been conclusively proven to be superior [61]. The optimal treatment should be individualized, taking into account factors such as life expectancy, PSA doubling time, Gleason score, and TNM stage.

In our review, most studies on salvage HIFU for radiation therapy-recurrent PCa employed a whole-gland ablation strategy. Based on prior experiences with salvage radiation therapy, a post-treatment PSA nadir of <0.5 ng/mL is considered a favorable indicator [62]. Similar findings were observed in the published literature [50]. Large-scale studies [53,54] demonstrated that whole-gland ablation effectively suppressed the median PSA nadir below 0.5 ng/mL, whereas smaller clinical trials employing hemigland or focal ablation failed to achieve this [51,52]. However, when comparing the 2-year progression-free survival between whole-gland and hemigland ablation [52,54], the former did not show a statistically significant advantage over the latter. This potential false-negative result highlights the heterogeneity in the current literature, likely due to the low statistical power of the published clinical trials.

While salvage RP after radiation therapy offers superior oncological control compared to salvage HIFU, it is associated with poor functional outcomes. According to the literature [63], continence is preserved in only up to 50% of men undergoing salvage RP, while potency recovery is even worse, with only 20–40% of men retaining erectile function. In contrast, the long-term pad-free continence rates after salvage HIFU range from 50% to 87%. In conclusion, salvage HIFU represents a treatment option that balances oncological and functional outcomes. Its primary use is in cases of local recurrence after radiation therapy, whereas its utility in local recurrence after RP is less well-documented in the current literature [64].

### 4.2. Combining HIFU with AS: Shaping a New Treatment Paradigm in Localized PCa

Applying HIFU in localized PCa has attracted the interest of urologists for years, and HIFU remains the most popular focal therapy in both published and ongoing studies [65]. The most appealing aspect of HIFU is its ability to preserve functional outcomes. In this review, we found that the 4-year incontinence rate and erectile dysfunction rate were approximately 3% and 30%, respectively. After RARP, the reported 12-month potency rates varied from 50% to 90%, while the continence recovery rate at 24 months after RP was about 83% in nerve-sparing cases and 45% in non-nerve-sparing cases [66]. Among these factors, HIFU showed the greatest advantage in continence recovery, although the incidence of new-onset erectile dysfunction after HIFU could still be as high as 40%.

In the latest version of the NCCN guidelines [4], radical treatment is excluded for certain localized PCa cases, such as very low-risk or low-risk cases with a life expectancy of less than 10 years. Although AS and observation are recommended with a high level of evidence in the guidelines, real world data show that approximately 30% of patients may opt for radical treatment due to poor compliance with surveillance protocols [3]. In a 15-year study [5], 54.8% of men choosing AS transitioned to radical treatment (RP or radiation therapy) within 10 years, with an additional 6.3% converting in the subsequent 5 years. This highlights a gap between AS/observation and radical treatment. Focal therapy, aiming to balance oncological and functional outcomes, has been proposed as an experimental option under concepts such as AS+ [67]. AS+ enhances the traditional AS protocol by incorporating focal therapy. Advanced technologies, such as 3T MRI, now enable the precise identification of index cancer within the prostate in its early stages [68], making localized ablation feasible.

By integrating HIFU into AS, researchers aim to achieve favorable oncological outcomes, including low-positive biopsy rates, reduced conversion rates to radical treatment, a metastasis rate of <5% at 10 years, and a PCa-specific mortality rate of <1% at 10 years [67]. In a previously published cohort study [5], more than 50% of men in AS transitioned to radical treatment within 10 years. By comparison, AS+ is estimated to improve this figure by approximately 50% [67]. For PCa-specific mortality, the published rate in AS was 2.2% at 10 years [5], whereas AS+ is projected to reduce this to 1% [67]. The metastasis rates in AS (7.1%) and AS+ (5%) showed lesser striking differences. Overall, this concept sheds light on the evolving role of HIFU and offers a feasible avenue for urologists to incorporate HIFU into the management of localized PCa.

There remains the possibility that men undergoing primary HIFU may eventually require salvage radical treatment, such as RP or radiation therapy. Historically, salvage RP after radiation therapy for localized PCa significantly compromises functional outcomes [68]. However, salvage RP after HIFU may present a different scenario. First, previous HIFU treatment does not appear to elongate the operation time [47]. Second, while incontinence rates after salvage RP following radiation therapy can be as high as 75% [69], continence rates after salvage RP following HIFU are comparable to those seen after primary RP [47]. Similar findings were observed for erectile function, suggesting that HIFU does not substantially impair functional outcomes in patients requiring salvage RP. According to the most statistically powered study to date [26], 15% to 40% of men, regardless of risk group, experience BCR within 5 years of HIFU. Offering salvage RP to these patients appears less concerning regarding functional outcomes, but further high-quality studies are needed to validate this hypothesis. Interestingly, the median and interquartile time to salvage RP after HIFU was less than 1.5 years, much shorter than the typical 5-year timeframe. This suggests that men requiring salvage RP after HIFU often exhibit early signs of recurrence. Similarly, among RARP patients with adverse features such as pT2+ disease, BCR is more likely to occur in the early postoperative period rather than later [70]. Identifying the factors associated with early recurrence after HIFU may help refine HIFU-based protocols, such as AS+, and improve treatment outcomes.

### 4.3. The Support of Using HIFU in Localized PCa: From Microscopic Viewpoints

After HIFU treatment of the prostate, one of the most prominent changes observed is microvascular alteration. The reduction in circulation following ablation induces granulomatous inflammation, necrosis, and fibrosis within the prostate [71]. In the microenvironment, the vascular structures become abnormal, characterized by malformed and atypical endothelium [72]. Studies have shown that angiogenesis is associated with tumor grade, progression, and metastasis in PCa [73]. In terms of prognosis, angiogenesis may carry different implications, depending on whether it occurs in tumoral or non-tumoral regions. One study observed a negative correlation between tumoral microcirculatory density and PCa recurrence, whereas a positive correlation was noted between non-tumoral microcirculatory density and PCa relapse [74]. Additionally, the study found that HIFU significantly increased the tumoral microcirculatory density. These findings suggest potential benefits of applying neoadjuvant ADT prior to HIFU [74].

Despite the current studies documenting the use of ADT in conjunction with HIFU, no subgroup analyses have been conducted to compare the oncological outcomes associated with this approach.

### 4.4. What Kind of Localized PCa Could HIFU Have Considered?

Localized PCa can be categorized into different risk groups based on PSA levels, imaging findings, and biopsy results. In pre-HIFU biopsy procedures, the transrectal and transperineal methods were the two main approaches. The former was easier to perform and had lower economic costs, while the latter provided better detection of cancer located in the apex and anterior zones, along with a lower risk of perioperative urosepsis [75]. A large-scale cohort study revealed that no cases of urosepsis occurred even with a 24-core fusion biopsy [76]. When determining which localized PCa risk groups are suitable for HIFU treatment, no strict selection criteria have been established. According to the clinical trials summarized in Table 2, most patients belonged to the D’Amico low- and intermediate-risk groups. However, PCa patients face a certain risk of lymph node (LN) involvement, which plays a crucial role in prognosis. Studies have demonstrated that patients with an intermediate risk or higher may have occult LN involvement when assessed using conventional hematoxylin and eosin staining, leading to a higher risk of biochemical recurrence (BCR) and poorer metastasis-free survival [77]. Therefore, patients undergoing HIFU need to be informed that an uncertain LN status poses a potential risk for future recurrence, and adherence to post-HIFU surveillance is essential.

When considering operational devices, tumoral regions at the posterior site, targets within 30 mm of the rectal wall, and prostate volumes less than 40 cc are conditions that facilitate the performance of HIFU. With advancements in technology, an increasing number of devices, such as 3T MRI and PSMA PET/CT, can assist in localizing tumoral regions within the prostate. A semiquantitative measurement called standardized uptake value (SUV) on PET/CT reflects PSMA expression, which is thought to correlate with tumor aggressiveness. In diagnostics, a SUV cut-off of 8 on 68Ga PSMA PET/CT has been found useful for predicting clinically significant PCa [78]. Therefore, incorporating PSMA PET/CT into the preoperative assessment can improve the localization of clinically significant PCa.

It has become trendy to adopt focal or hemigland ablation for treating localized PCa, but high-quality clinical trials supporting these two methods remain scarce. According to the largest clinical study in the United States [79], which included 17% intermediate-unfavorable risk and 5% high-risk patients [4], 91% of radical treatments could be avoided, and 73% of patients maintained successful treatment outcomes at 2 years. In their report, approximately 70% of the included cases had localized PCa greater than GG2 at baseline, but only 24% showed recurrent cancer greater than GG2 at 2 years [79]. From this perspective, the oncological outcomes after hemigland HIFU appear promising. However, it is worth noting that the post-HIFU PSA nadir for focal and hemigland ablation tends to be higher than that observed in patients undergoing whole-gland ablation, as shown in Table 2. In one study using 3T MRI and transperineal template-guided mapping biopsy (mean biopsy cores: 29), the sensitivity, specificity, and accuracy in determining eligibility for hemigland ablation were 80.7%, 85.1%, and 82.5%, respectively. However, the risk of undetected contralateral clinically significant PCa was as high as 19.2% [80].

A retrospective study [81] indicated that post-HIFU PSA nadir could be a meaningful predictor of oncological outcomes. After whole-gland ablation, a PSA nadir below 0.2 was associated with better BCR-free survival. Therefore, patients opting for focal or hemigland ablation should undergo post-HIFU biopsy or imaging to assess their cancer status and discuss the potential need for adjuvant or salvage treatment. Among men receiving focal ablation, approximately 30% had positive biopsies on the treated side 6 months after HIFU, and half of these cases involved clinically significant cancer. Interestingly, around 7% of patients had positive biopsies on the untreated side, and half of these also involved clinically significant cancer [56]. In cases of positive biopsy following HIFU, patients often preferred to receive ADT as salvage treatment [34]. For high-risk localized PCa, whole-gland ablation could be considered when the potential benefits of HIFU outweigh the risks, particularly in situations where radical treatment is unsuitable [82]. It is worth noting that both continence and erectile function recovery were excellent after hemigland ablation [79].

### 4.5. After HIFU, What Should We Do?

In addition to focal and hemigland ablations, expert consensus provides valuable guidance for clinical care following whole-gland ablation [82]. For postoperative surveillance, the consensus recommends combining PSA testing with a digital rectal examination (DRE). PSA should ideally be monitored every 3 to 6 months during the first 5 years after HIFU and then annually thereafter. Additionally, 3T MRI may be considered at 6 or 12 months after whole-gland ablation to assess the disease status. To date, the surveillance protocol after whole-gland ablation closely resembles that of active surveillance (AS). Furthermore, experts recommend imaging—preferably 3T MRI—before performing a biopsy in patients who meet the criteria for biochemical recurrence (BCR). If a biopsy is deemed necessary, it should ideally combine targeted and systematic approaches for optimal accuracy.

According to expert opinion [82], PSMA PET/CT (18F/68Ga) can be utilized in cases of post-HIFU recurrence. Its role includes staging positive recurrent cancer biopsies and detecting suspicious metastases in cases of negative biopsy results. The adoption of PSMA PET/CT in post-HIFU surveillance could be a game changer [83], as false-negative readings from post-HIFU 3T MRI are not uncommon. Preliminary data have shown that 68Ga PSMA PET/CT can effectively detect recurrence that is occult on 3T MRI, particularly in patients with GG group ≥ 3 [84]. With its advantages of a lower infection rate and better prostate sampling, transperineal biopsy is becoming increasingly popular. One large-scale study demonstrated that the biopsy method initially adopted influences the likelihood of a subsequent re-biopsy [85]. Given that transperineal biopsy significantly enhances cancer detection, it should be the preferred method for assessment. In another study assessing transperineal transrectal ultrasound-guided biopsy [86], no significant differences in postoperative complications—such as hematuria, acute urinary retention, and hematospermia—were observed between the 12-core and 18-core biopsies. However, the 18-core biopsy showed a higher detection rate in the early phase, specifically in cases with PSA < 10 ng/mL and negative DRE.

Regarding the definition of BCR, experts recommend using the Phoenix criteria [81]. Among the current literature, not only are post-HIFU imaging surveys and biopsy methods inconsistent, but the definition of BCR also varies across studies. In the clinical trials we reviewed, most used the Phoenix criteria to define BCR. However, this lack of standardization can lead to heterogeneity when assessing post-HIFU-positive biopsy rates and BCR-free survival. An external validation study suggested that using the criteria reported by Huber et al.—PSA nadir +1 ng/mL in the first year and PSA nadir +1.5 ng/mL in the second year—might be more accurate in detecting BCR [87]. Beyond the definition of BCR, other sources of heterogeneity arise from surveillance methods. For example, some studies performed an immediate biopsy after HIFU while others did not. Similarly, some studies included 3T MRI in post-HIFU surveillance, with varying timelines for the first imaging—some at 6 months and others at 12 months post-HIFU. These inconsistencies contribute to uncertainty about the residual cancer status. In a study of hemigland ablation, a positive biopsy was found in as many as 25% of treated lobes and in approximately 35% of untreated lobes [30], suggesting that a notable proportion of cancer may persist after HIFU.

In the United States, the economic burden of prostate cancer treatment has placed significant strain on the healthcare system. A deterministic decision analytic model estimated that adopting active surveillance (AS) for low-risk localized PCa could save approximately 16,000 USD per patient over a 10-year period [88]. As a result, AS combined with other prognostic tools is considered a potential solution to alleviate the economic burden. In the United Kingdom, studies have shown that the overall cost of HIFU is significantly lower than prostatectomy and radiation therapy. A Markov cohort health state transition model over a 10-year period demonstrated that HIFU not only reduced costs but also significantly improved quality-adjusted life years (QALYs) [89]. Thus, the concept of AS+ holds promise for reducing medical expenses while maintaining or improving patient outcomes in the future.

## 5. Conclusions

According to the NCCN guidelines, HIFU can be offered as a treatment option for non-metastatic recurrent PCa following radiation therapy. In the case of localized PCa, HIFU is the most promising among all focal therapy options; however, it is not yet officially recommended for any risk groups in the guidelines. Nonetheless, it is considered legitimate to offer HIFU to patients in low- and intermediate-risk groups based on expert consensus. Regarding oncological outcomes, no high-quality clinical trials are currently available for reference. Among the published literature supporting HIFU, the recovery of continence remains its most appealing benefit. To reduce heterogeneity, future clinical trials should aim to standardize the definition of BCR and unify the imaging devices and biopsy methods used in surveillance. In ongoing clinical trials, combining AS with HIFU appears to be a promising approach, as it may significantly reduce the conversion rate to radical treatment. However, the actual treatment effects have not yet been reported.

Based on published clinical trials, we recommend whole-gland ablation as the general approach, as it provides a lower PSA nadir and avoids the higher positive biopsy rates observed after focal and hemigland ablation in both treated and untreated lobes. For cases where focal or hemigland ablation is necessary, careful patient assessment and detailed explanations regarding the potential for post-HIFU-positive biopsies should be provided, as these are associated with inferior oncological outcomes, including a lower BCR-free survival rate. For post-HIFU surveillance, we suggest conducting 3T MRI with a biopsy at 6 to 12 months after treatment to reassess the cancer status. The Phoenix criteria should be adopted to define BCR. In cases of suspected recurrence despite negative biopsy results, PSMA PET/CT can be used to identify recurrent foci.

## Figures and Tables

**Table 1:** HIFU in salvage therapy against prostate cancer recurrence.

	Device and Ablative Method	PSA Level When Receiving HIFU	Follow-Up	Oncological Outcome	Functional Outcome	Complications
Siddiqui et al. [50] *N* = 81	Sonablate^®^: whole-gland salvage therapy	Mean ± SD ^a^: 4.06 ± 2.88 ng/mL	Mean ± SD ^a^: 53.5 ± 31.6 months	Cancer free at 6 months after HIFU: 65% 5-y ^b^ overall survival: 88% Progression to androgen deprivation therapy: 26.9% Mean time to androgen deprivation therapy: 26.14 months	IPSS ^c^:↑within 45 days; ↓after 45 days IIEF ^d^-5 at 6 months: ↓ Incontinence at 6 months: 3.7%	Severe rectal fistula: 3.7% Side effects of Clavien-Dindo above III within 180 days: 6 (7.4%)
Ahmed et al. [51] *N* = 39	Sonablate^®^: focal salvage therapy	Median: 3.3 ng/mL	Median: 17 (IQR ^e^: 10–29) months	PSA nadir: median 0.57 (IQR ^e^: 0.1–2.3) ng/mL Mean time to PSA nadir: 4.3 months 1-y ^b^ BCR ^f^-free survival: 55% 2-y ^b^ BCR ^f^-free survival: 24%	Pad-free: 87% IIEF ^d^-5 at 6 months: ↓	Side effects of Clavien-Dindo above III: 10 (25.6%)
Baco et al. [52] *N* = 48	Ablatherm^®^: hemigland salvage therapy	Not provided	Median: 16.3 (IQR ^e^: 10.5–24.5) months	Mean PSA nadir: 0.69 ng/mL Time to PSA nadir: 21 weeks 1-y ^b^ PFS ^g^: 83% 18-month PFS ^g^: 64% 2-y ^b^ PFS ^g^: 52%	IPSS ^c^: ↓ IIEF ^d^-5:↓ Pad-free: 75%	2 (4.1%) pubic bone osteitis and 1 of them (2.0%) pubovesical fistula; no Clavien-Dindo grade was reported
Crouzet et al. [53] *N* = 290	Ablatherm^®^: whole-gland salvage therapy	Mean ± SD ^a^: 6.38 ± 7.61 ng/mL	27–48 months	Median PSA nadir: 0.14 ng/mL Mean time to PSA nadir: 5.55 months 7-y ^b^ CSS ^h^: 80% 7-y ^b^ MFS ^i^: 79.6%	Long-term pad-free: 50%	Bladder outlet obstruction: 16% Urinary diversion for recurrent stricture: 1.3% Ureterorectal fistula: 2% Anal incontinence: 0.7% Pubic bone osteitis: 2.7%
Uddin Ahmed et al. [54] *N* = 86	Sonablate^®^: whole-gland salvage therapy	Median: 5.7 (IQR ^e^: 1.5–7.7) ng/mL	Mean: 19.8 months	Median PSA nadir: 0.2 ng/mL Mean time to PSA nadir: 3.1 months 1-y ^b^ PFS ^g^: 59% 2-y ^b^ PFS ^g^: 43%	IPSS ^c^:↑ at 6 months IIEF ^d^-5: ↓ at 6 months Pad-free: 62%	Post-procedure urinary tract infection/epididymitis: 29% Pubic bone osteitis: 1.2% Ureterorectal fistula: 2.4%
Palermo et al. [55] *N* = 22	Brand of HIFU device not mentioned; whole-gland salvage therapy	Median: 1.27 (range: 0.3–8) ng/mL	Median: 48 (28–72) months	45.5% had PSA ≤ 0.4 ng/mL at 3 months No cancer-related death in study period 23% needed total androgen blockade in study period	De novo erectile dysfunction at 3 months: 28.5% De novo severe incontinence requiring surgery: 5.8%	Ureteral/anastomosis stricture: 4.5% (Clavien-Dindo IIIb) Acute urine retention: 4.5%

^a^ Standard deviation; ^b^ Year; ^c^ International Prostate Symptom Score; ^d^ International Index of Erectile Function; ^e^ Interquartile range; ^f^ Biochemical recurrence; ^g^ Progression-free survival; ^h^ Cancer-specific survival; ^i^ Metastasis-free survival.

**Table 2:** HIFU therapy in localized prostate cancer.

	Device and Ablative Method	Age and Follow-Up	PSA	Gleason Grade Group	T-Stage	D’ Amico Risk Group	Oncological Outcome	**Functional Outcome**
Crouzet et al. [26] *N* = 1002	Ablatherm^®^: whole-gland ablation	Median age: 71 (range: 48–87) yrs ^a^ old Median f follow-up: 6.4 yrs (range: 0.2–13.9).	Median: 7.7 (range: 0–30) ng/mL	GG ^b^ 1: 55.4% GG ^b^ 2/3:34.7% Above GG ^b^ 4: 8.4%	T1: 51.7% T2:44.8% T3: 2.8%	Low: 35.6% Intermediate: 45.1% High: 17.4%	All reach PSA nadir within 6 months: median 7.9 wks Median PSA nadir: 0.14 (range: 0–12.7) ng/mL 5-y ^a^ BCR ^c^-free for low risk: 76–86% 5-y ^a^ BCR ^c^ free for intermediate risk: 63–78% 5-y ^a^ BCR ^c^ free for high risk: 57–68% 10-y ^a^ OS ^d^: 80% 10-y ^a^ CSS ^e^: 97% 10-y ^a^ MFS ^f^:94% 10-y ^a^ MFS ^f^ for low risk: 99% 10-y ^a^ MFS ^f^ for intermediate risk: 95%	Severe incontinence: 5% Erectile function preservation: 42.3%
Gelet et al. [28] *N* = 82	Ablatherm^®^: whole-gland ablation	Mean age: 71 (SD ^g^: 5.7) yrs ^a^ Mean follow-up: 17.6 (range: 3–68.5) mo ^h^	Mean: 8.11 (SD ^g^: 4.64) ng/mL	GG ^b^ 1: 58.5% GG ^b^ 2/3: 25.6% Above GG ^b^ 4: 15.9%	T1: 46.3% T2:48.8% Local recurrence after EBRT ^i^: 4.9%	Low risk: 39.1% Intermediate risk: 60.9%	Mean PSA nadir: 0.63 ng/mL 78% no cancerous foci after HIFU 5-y ^a^ PFS ^j^: 62%	De novo impotence: 77% De novo incontinence 17%:
van Velthoven et al. [29] *N* = 50	Ablatherm^®^: hemigland ablation	Median Age: 73 (IQR ^k^: 70–77) yrs ^a^ Median follow-up: 34 (IQR ^k^: 13–58) mo ^h^	Median: 6.3 (IQR ^k^:3.9–8.3) ng/mL	GG ^b^ 1: 60% GG ^b^ 2: 28% GG ^b^ 3:12%	T1: 32% T2: 68%	Low risk: 48% Intermediate risk: 52%	Median PSA nadir: 0.91 (IQR: 0.52–2.07) ng/mL Median time to PSA nadir: 3 (1–6) mo 5-y ^a^ RFS ^l^: 58% 5-y ^a^ CSS ^e^: 100% 5-y ^a^ MFS ^f^: 93% 5-y ^a^ OS ^d^: 87%	De novo impotence: 20% Persistent incontinence: 6%
Ganzer et al. [30] *N* = 54	Ablatherm^®^: hemigland ablation	Mean age: 63.4 (SD ^g^: 8.3) yrs ^a^ Mean follow-up: 17.41 (SD ^g^: 4.5) mo ^h^	Mean: 6.2 (SD ^g^: 2.1) ng/mL	GG ^b^ 2: 15.7% Other GG ^b^s not mentioned	Not mentioned	All were low and intermediate; but no proportion was reported	Mean PSA nadir: 2.9 ng/mL Positive biopsy at the treated lobe after HIFU: 26.5% Positive biopsy at the contra-lateral lobe after HIFU: 34.7% Positive biopsy at the bilateral lobes after HIFU: 6.1%	De novo impotence: 30% De novo incontinence: 4.9%
Muto et al. [34] *N* = 70	Sonablate^®^: both whole-gland and focal ablation (whole-gland ablation: 58.6%; focal ablation: 41.4%)	Median age: 72 (range: 61–80) yr ^a^ Median follow-up: 34 (SD ^g^: 8–45) mo ^h^	Median: 4.6 (range: 0–29.5) ng/mL	GG ^b^ 1: 52.9% GG ^b^ 2/3: 30% Above GG ^b^ 4: 11.4% Unknown: 5.7%	T1:81.4% T2a: 11.4% T2b: 7.1%	Not mentioned	Mean PSA nadir after whole-gland ablation (categorized according to timeline): 1.14 (SD ^g^: 1.58) ng/mL Mean PSA nadir after focal ablation (categorized according to timeline): 2.74 (SD ^g^: 3.37) ng/mL 2-y ^a^ BCR ^c^-free survival (low risk; whole-gland ablation): 85.9% 2-y ^a^ BCR ^c^-free survival (low risk; focal ablation): 49.9% 2-y ^a^ BCR ^c^-free survival (intermediate; whole-gland ablation): 53.6% 2-y ^a^ BCR ^c^-free survival (high): 0%	Not mentioned
Uchida et al. [35] *N* = 72	Sonablate^®^: whole-gland ablation	Median age: 72 (range: 45–79) yrs ^a^ Median follow-up: 14 (range: 2–24) mo ^h^	Median: 8.1 (range: 2.1–19.8) ng/mL	Above GG ^b^ 4: 8%	T1: 56% T2a: 25% T2b: 19%	Not mentioned	2-y ^a^ BCR ^c^-free survival: 76%	De novo impotence: 38% De novo incontinence: 1%
Shoji et al. [41] *N* = 326	Sonablate^®^: whole-gland ablation	Mean age: 68 (range:45–88) yrs ^a^ Follow-up: not mentioned	Mean PSA: 12.7 (range: 3.39–69.4) ng/mL	Above GG ^b^ 4: 11.6%	T1: 53% T2a: 32.6% T2b:14.4%	Not mentioned	8-y ^a^ BCR ^c^-free survival for the low risk: 84% 8-y ^a^ BCR ^c^-free survival for the intermediate risk: 64% 8-y ^a^ BCR ^c^-free survival for the high risk: 45%	IIEF ^m^-5 at 24 mo ^h^ after HIFU: no significant change Incontinence: not reported
Duwe et al. [46] *N* = 29	Ablatherm^®^: focal and hemi ablation	Median age: 66 (IQR ^k^: 61.5–72.5) yrs ^a^ Median follow-up: 23 mo ^h^	Median PSA: 6.8 (IQR ^k^: 5.1–8.9) ng/mL	GG ^b^ 1: 69% GG ^b^ 2: 27.6% GG ^b^ 3: 3.45%	All were judged to be unilateral cancer before HIFU	Not mentioned	Median time to PSA nadir: 6 mo ^h^ 2-y ^a^ RFS ^l^: 62%	4-y ^a^ erectile dysfunction: 31% 4-y ^a^ incontinence: 3%
Ahmed et al. [56] *N* = 56	Sonablate^®^: focal or hemi ablation	Mean age: 63.9 (SD ^g^: 5.8) yrs ^a^ Follow-up: 12 mo ^h^	Median PSA: 7.4 (IQR ^k^: 5.6–9.5) ng/mL	No patients above GG ^b^ 4	T1: 28.6% T2a: 16.1% T2b:32.1% T2c: 19.6% T3a:3.6%	Low risk: 12.5% Intermediate risk: 83.9% High risk: 3.6%	Median PSA nadir: 2.4 (IQR ^k^: 1.6–4.1) ng/mL 1-y ^a^ absence of clinically significant PCa ^n^: 80.8% 1-y ^a^ absence of measurable PCa ^n^: 85.7%	1-y ^a^ leak-free continence: 92% 1-y ^a^ erectile function recover: 77%

^a^ year(s); ^b^ Gleason grade; ^c^ Biochemical recurrence; ^d^ Overall survival; ^e^ Cancer-specific survival; ^f^ Metastasis-free survival; ^g^ Standard deviation; ^h^ month(s); ^i^ External beam radiation therapy; ^j^ Progression-free survival; ^k^ Interquartile range; ^l^ Recurrence-free survival; ^m^ International Index of Erectile Function; ^n^ Prostate cancer.

**Table 3:** Salvage radical prostatectomy after HIFU therapy.

	Operative Method	Age and the Time Between HIFU and Salvage RP	Risk Group at HIFU and PSA Before Salvage RP	Operative Time	Oncological Outcome	Functional Outcome	Complications
Spitznagel et al. [47] *N* = 39 (salvage RP: 13; primary RP: 26)	Robot-assisted; DaVinci system (Intuitive Surgical Inc., Sunnyvale, CA, USA)	The median age: 61.0 (IQR ^a^: 58.0–66.0) yrs ^b^ The median time to salvage RP: 14.5 (IQR: 11–15) mo	Risk group at HIFU: 10 intermediate and 3 high risk group in salvage RP ^c^ PSA before salvage RP ^c^: median 5.4 (4.6–7.8) ng/mL	Salvage RP ^c^: 260 (IQR ^a^: 232–275) minutes Primary RP ^c^: 257 (IQR: 228–283) minutes	PSM ^d^: 1/13 (7.6%) BCR ^e^ at 12 mo ^f^ after salvage RP ^c^: 1/13 (7.6%)	Continence and erectile function of salvage RP ^c^ were not significantly different from the primary RP ^c^.	Prolong catheter indwelling (Clavien-Dindo class I) Anastomosis leakage with peritonitis (Clavien-Dindo class IIIa) Lymphocele with infection (Clavien-Dindo class IIIa) Lymphedema of scrotum and ascites (Clavien-Dindo class IIIa) Fascia dehiscence (Clavien-Dindo class IIIb)

^a^ Interquartile range; ^b^ year(s); ^c^ Radical prostatectomy; ^d^ Positive surgical margin; ^e^ Biochemical recurrence; ^f^ month(s).

## Data Availability

Not applicable.
